# Prognostic significance of microRNA miR-24 in cancers: a meta-analysis

**DOI:** 10.1080/21655979.2021.1875662

**Published:** 2021-02-08

**Authors:** Rongqiang Liu, Weihao Kong, Shiyang Zheng, Chenyu Yu, Yajie Yu, Yuling Xu, Linsen Ye, Yi Shao

**Affiliations:** a Department of Ophthalmology, The First Affiliated Hospital of Nanchang University, Nanchang, Jiangxi, China; b Department of Hepatobiliary Surgery, The First Affiliated Hospital of Guangzhou Medical University, Guangzhou, Guangdong, China; c Department of Emergency Surgery, Department of Emergency Medicine, The First Affiliated Hospital of Anhui Medical University, Hefei, Anhui, China; d Department of Breast Surgery, The Third Affiliated Hospital of Guangzhou Medical University, Guangzhou, China; e Department of Hepatic Surgery and Liver Transplantation Center, The Third Affiliated Hospital of Sun Yat-sen University, Guangzhou, China

**Keywords:** mir-24, prognosis, cancer, meta-analysis

## Abstract

The prognostic significance of miR-24 in tumors has not been determined. Therefore, we conducted a meta-analysis to systematically assess the correlation between miR-24 and its prognostic value in cancers PubMed, EMBASE, and Web of Science databases were used to search relevant articles (up to 1 October 2020). Studies that evaluated the prognostic value of miR-24 in tumors were included. The hazard ratio (HR) and odds ratio (OR) with 95% confidence intervals (CI) were used to evaluate survival outcomes and clinical characteristics. All data analyses were implemented using STATA 12.0 software. A total of 17 studies from 15 articles involving 1705 patients were collected for the meta-analysis. The pooled analysis revealed that elevated miR-24 expression was obviously associated with poor overall survival (OS) (HR = 1.66, 95% CI: 1.20–2.31). Furthermore, we also found that elevated miR-24 expression was positively correlated with tumor size (large or small) and tumor stage (III–IV vs I–II). Elevated miR-24 expression indicates poor prognosis and may be a promising prognostic marker in different cancers. Our findings needed to be verified through further investigations.

**Figure:**
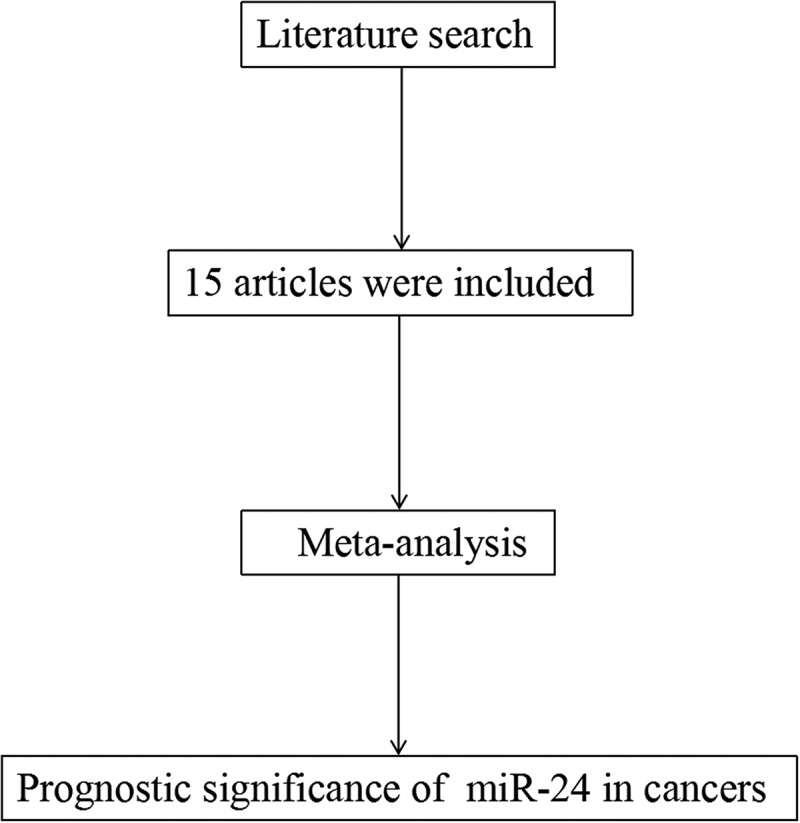

## Introduction

In recent years, cancer incidence has been on the rise, and studies investigating the molecular mechanisms involved have intensified. MicroRNA (miRNA) is considered to be closely associated with tumor progression. These small-molecule miRNAs are not directly involved in protein synthesis, but can affect mRNA-mediated gene expression by binding to the 3ʹ non-coding region of specific mRNA. Abnormal regulation of the expression of proto-oncogenes and tumor suppressor genes by miRNA plays an important part in tumorigenesis. Accumulated evidence discloses that miRNAs are indispensable in cancer progression and many different miRNAs have been applied to tumor diagnosis and prognosis [1,2].

MiR-24 is an important member of the miRNA family. It is located in two different gene clusters. One is the miR-23b/miR-27b/miR-24-1 gene cluster, located in the expression sequence region of chromosome 9 [3]. The other gene cluster is miR-23a/miR-27a/miR-24-2, located in the intergenic region of chromosome 19 [4]. Both share a common activated region miR-24-3p. MiR-24 is considered as a tumor-promoting factor and is abnormally expressed in many tumors. Studies have revealed that miR-24 involves in tumor cell proliferation, differentiation, invasion, and metastasis by regulating numerous genes, such as PTEN, TRIM11, SOX7, PRKCH, and P16 [5–9]. Recently, abnormal miR-24 expression has been reported to be correlated with the prognosis and clinical characteristics in tumors, including gastric cancer (GC), lung cancer (LC), colorectal cancer (CRC), acute lymphoblastic leukemia (ALL), acute myeloid leukemia (AML), hepatocellular carcinoma (HCC), nasopharyngeal carcinoma (NPC), and tongue squamous cell carcinoma (TSCC) [10–24]. Most studies reported that high miR-24 expression predicted poor prognosis, while several other studies shown the opposite view. The results are controversial.

Nowadays, the prognostic value of miR-24 in tumors is not clear. Therefore, in this study, we have summarized the existing literatures and conducted a meta-analysis to comprehensively assess the prognostic significance of miR-24 in tumors.

## Methods

### Literature search

PubMed, Web of Science and EMBASE were used to conduct comprehensive search for eligible studies (up to 1 October 2020). The following terms were applied for the search strategy: ‘miR-24’ or ‘microRNA-24’ or ‘miRNA-24’ and ‘cancer’ or ‘carcinoma’ or ‘tumor’ or ‘tumour’ or ‘neoplasm’ and ‘prognosis’ or ‘survival’ or ‘prognostic’ or ‘outcome’. No limitation on language was applied to retrieve publications.

### Study selection

The inclusion criteria for the studies were: 1) patients enrolled were pathologically diagnosed; 2) the expression of miR-24 and the prognosis outcomes of cancer patients were investigated; 3) the hazard ratio (HRs) with their 95% confidence interval (CI) of survival outcomes were directly reported or could be indirectly extracted from survival curves. The exclusion criteria were: 1) publications that were reviews, letters, meeting abstracts, animal studies, and non-comparative studies; 2) studies focusing on polymorphisms or methylation patterns of miR-24; 3) studies with insufficient data to retrieve HRs with CIs of survival outcomes; and 4) duplicated publications.

### Data extraction and quality assessment

Two independent investigators extracted all data, and the third investigator resolved any disagreements. The following information was collected from the enrolled studies: the name of first author, publication country, publication year, tumor type, number of patients, miRNA type, sample source for miRNA detection, detection method, HRs with CIs of survival outcomes including overall survival (OS), disease-free survival (DFS), recurrence-free survival (RFS), and progression-free survival (PFS). For studies only providing survival curves, Tierney’s method was used to extract HRs and 95% CIs [25]. The quality of included studies was evaluated according to the Newcastle Ottawa scale (NOS) [26]. The NOS score ranges from 0 to 9, and studies with a NOS score >6 were defined as high quality.

### Statistical analysis

Pooled HRs with 95% CIs were applied to assess the influence of miR-24 expression on cancer prognosis. The heterogeneity of pooled HRs was estimated by Cochran’s Q test and Higgins I^2^ statistic [27]. P-value <0.05 or I^2^ > 50% was defined as significant heterogeneity, and the random-effects model was used to pool HRs [28]. For all other instances, the fixed-effect model was used. The stability of the pooled HRs was evaluated by sensitivity analysis. In addition, subgroup analyses were also conducted. Begg’s and Egger’s tests were used to evaluate the publication bias, and P-value <0.05 was considered as significant publication bias [29,30]. The trim-and-fill method was used to assess the robustness of the meta-analysis results in the presence of publication bias [31]. P < 0.05 is considered statistically significance [30]. All the statistical analyses for this meta-analysis were executed in STATA version 12.0 (Stata Corporation, College Station, TX, United States).

## Results

### Brief introduction

The prognostic value of miR-24 in tumors has not been determined. Therefore, we present a meta-analysis to clarify the prognostic value of miR24 in various cancers. First, we searched the designated database to find the literatures on the prognostic value of miR-24 in tumors, and then extracted relevant data for meta-analysis. We discovered that elevated miR-24 expression was significantly associated with OS, tumor size (large or small) and tumor stage (III–IV vs I–II).

### Literature selection

**Figure 1.**
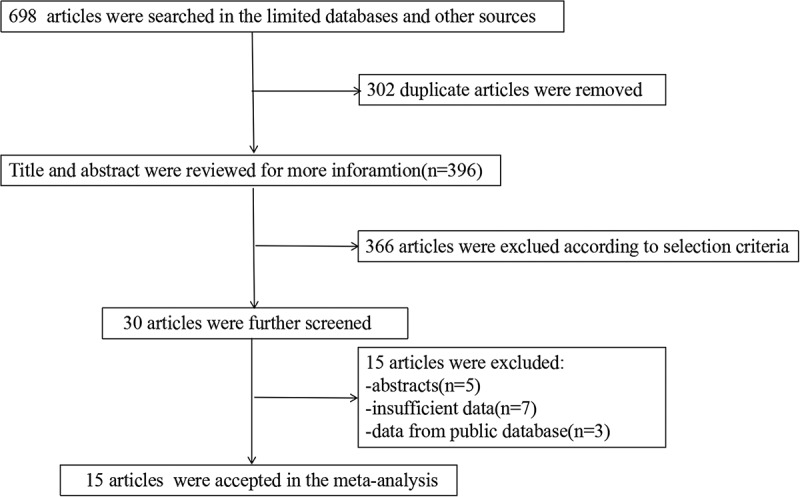
Flow chart of the literature search

Articles investigating the prognostic value of miR-24 in tumors were searched and retrieved from the electronic databases. Six hundred and ninety-eight articles were initially collected. After excluding 302 duplicates, 396 articles remained. After trimming 366 irrelevant articles, 30 articles were examined. After screening the full text of these articles, 15 articles were excluded. Eventually, 15 articles published between 2012 and 2018 were included in the study [10–24]. The study flow chart is shown in Figure 1.

### Study characteristics

A total of 17 studies from 15 articles involving 1705 patients were enrolled in the study. Fourteen studies reported OS data, and seven studies reported DFS/PFS/RFS. One article was from Mexica, and all other articles originated from China. Four articles reported survival results in the multivariate analysis and 11 articles performed univariate analysis. All articles used RT-qPCR to detect the expression of miR-24. Two studies tested the expression of miR-24 in serum, and 15 studies tested the expression of miR-24 in tissues. The tumor types included were GC [10,19], CRC [11,13], leukemia [12,20], TSCC [22,23], HCC [15,17], LC [21,24], NPC [18], and osteosarcoma [16]. The average NOS score result was 6.5. Basic information about the included studies is displayed in Table 1.

**Table 1.** The basic information of included studies

Study	Country	Tumor type	No. of patients	microRNA type	Detected sample	Detected method	Analysis type	Survival analysis	Source of HR	NOS score
Dong 2018	China	GC	247	miR‑24	Tissue specimens	RT-qPCR	Multivariate	OS	Reported	7
Gao 2015	China	CRC	175	miR‑24-3p	Tissue specimens	RT-qPCR	Multivariate	OS	Reported	7
Organista-Nava 2015A	Mexico	ALL	111	miR-24	Tissue specimens	RT-qPCR	Univaritae	OS	K-M curves	7
Organista-Nava 2015B	Mexico	AML	36	miR-24	Tissue specimens	RT-qPCR	Univaritae	OS	K-M curves	7
Kerimis 2017	Greece	CRC	182	miR‑24-3p	Tissue specimens	RT-qPCR	Multivariate	OS, DFS	Reported	7
Le 2012	China	LC	82	miR‑24	Serum	RT-qPCR	Multivariate	OS	Reported	7
Liu 2014	China	HCC	207	miR‑24	Tissue specimens	RT-qPCR	Univaritae	OS, RFS	Reported	7
Liu 2018	China	Osteosarcoma	84	miR‑24	Tissue specimens	RT-qPCR	Univaritae	OS	K-M curves	7
Meng 2014	China	HCC	72	miR‑24-3p	Tissue specimens	RT-qPCR	Multivariate	OS, DFS	Reported	7
Su 2018	China	NPC	23	miR‑24	Tissue specimens	RT-qPCR	Univaritae	PFS	K-M curves	6
Wu 2017	China	GC	28	miR‑24	Tissue specimens	RT-qPCR	Univaritae	OS	K-M curves	7
Yin 2015	China	AML	84	miR‑24	Tissue specimens	RT-qPCR	Univaritae	OS, RFS	K-M curves	7
Zhao 2015A	China	LC	53	miR‑24	Tissue specimens	RT-qPCR	Univaritae	RFS	K-M curves	6
Zhao 2015B	China	LC	67	miR‑24	Serum	RT-qPCR	Univaritae	RFS	K-M curves	6
Zhao 2016	China	TSCC	84	miR‑24	Tissue specimens	RT-qPCR	Univaritae	OS	K-M curves	6
Zhao 2017	China	TSCC	90	miR‑24	Tissue specimens	RT-qPCR	Multivariate	OS	K-M curves	7
Zhou 2018	China	LC	50	miR‑24	Tissue specimens	RT-qPCR	Univaritae	OS	Reported	7

Abbreviation: GC: gastric cancer; LC: lung cancer; CRC: colorectal cancer; ALL: acute lymphoblastic leukemia; AML: acute myeloid leukemia; HCC: hepatocellular carcinoma; NPC: nasopharyngeal carcinoma; TSCC: tongue squamous cell carcinoma.

### Association between elevated miR-24 expression and OS

**Figure 2.**
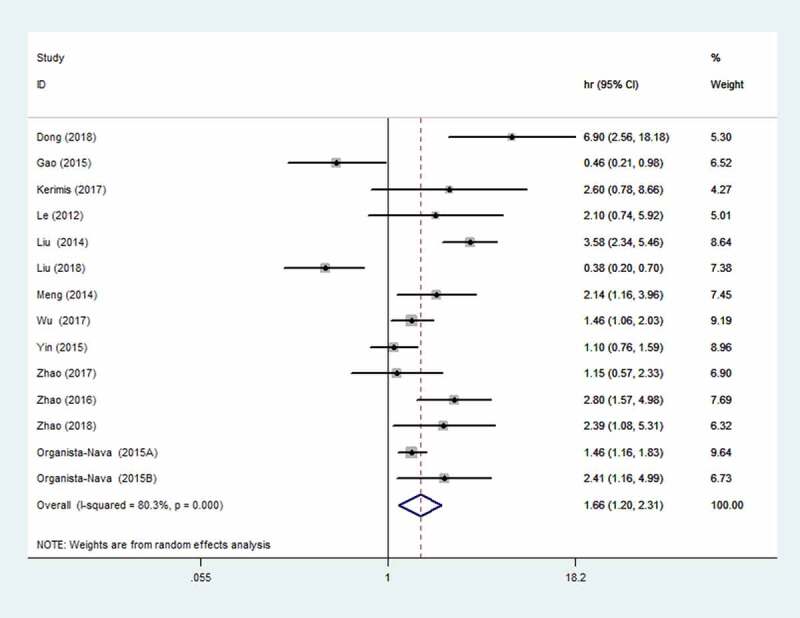
Forest plot of the association between elevated miR-24 expression and OS

Due to the significant heterogeneity (I^2^ = 80.3%), the random-effects model was used. The results indicated that elevated miR-24 expression was closely related to OS (Figure 2). Elevated miR-24 expression revealed poor OS (HR = 1.66, 95% CI: 1.20–2.31).

### Subgroup and regression analysis

In order to better detect the predictive value of miR-24, we performed subgroup analysis based on cancer type, analysis type, race, source of HR, sample size, detected sample, and miRNA type (Table 2). We observed a significant correlation between miR-24 and OS for the following subgroups: ALL, HCC, LC, univariate analysis, White race, reported, sample size <100, detection sample (tissue), and detection of miR-24. For other subgroups analyzed, no significant statistical differences were found.

**Table 2.** Subgroup analysis for OS

Stratifed analysis	No. of studies	Pooled HR (95%CI)	*P*-value	Heterogeneity		
				I2 (%)	*P*-value	Model
Cancer type						
GC	2	2.96 (0.65–13.49)	0.161	88.5	0.003	Random
CRC	2	1.02 (0.19–5.56)	0.981	82.5	0.017	Fixed
leukemia	3	1.40 (1.17–1.69)	0	48.8	0.142	Fixed
TSCC	2	1.84 (0.77–4.38)	0.167	72.6	0.056	Random
HCC	2	3.03 (2.14–4.30)	0	45.1	0.177	Fixed
LC	2	2.28 (1.21–4.29)	0.011	0	0.843	Fixed
Osteosarcoma	1	0.38 (0.20–0.70)				
Analysis type						
Univariate	8	1.62 (1.10–2.39)	0.015	84.3	0	Random
Multivariate	6	1.79 (0.87–3.65)	0.112	76.7	0.001	Random
Race						
Caucasian	3	1.55 (1.25–1.92)	0	16.3	0.303	Fixed
Asian	11	1.61 (1.04–2.49)	0.034	84.3	0	Random
Source of HR						
Reported	7	2.01 (1.07–3.78)	0.03	79.9	0	Random
SC	7	1.43 (0.99–2.05)	0.056	78.5	0	Fixed
Sample size						
≥100	5	2.04 (0.98–4.22)	0.055	88	0	Random
<100	9	1.50 (1.03–2.19)	0.034	73.7	0	Fixed
detected sample						
Tissue	13	1.64 (1.17–2.31)	0.004	82.3	0	Random
Serum	1	2.095 (0.741–5.923)				
miRNA type						
miR-24	11	1.76 (1.23–2.50)	0.002	81.6	0	Random
miR-24-3p	3	1.32 (0.43–4.05)	0.629	81.8	0	Random

### Association between elevated miR-24 expression and DFS/PFS/RFS

**Figure 3.**
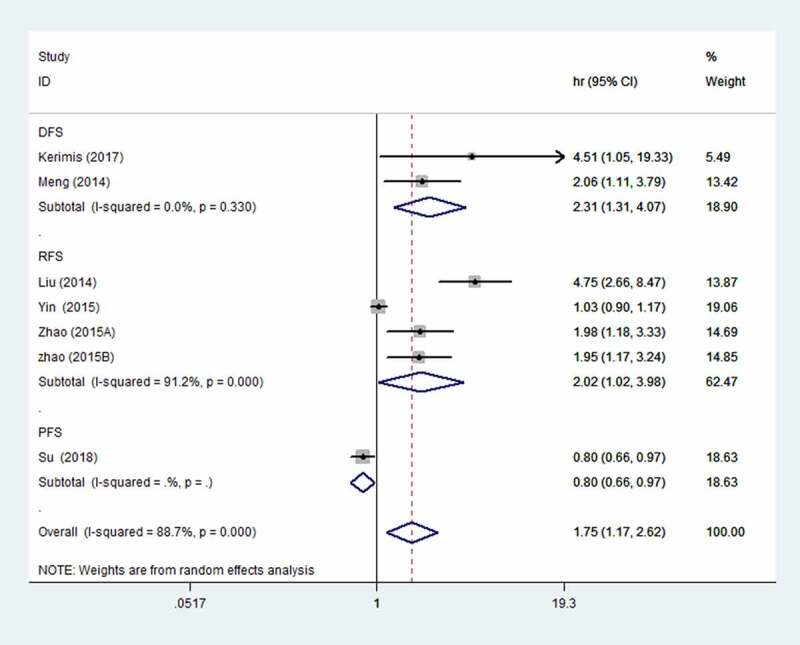
Forest plot of the association between elevated miR-24 expression and DFS/PFS/RFS

Seven studies involving 688 patients reported DFS/PFS/RFS. The result revealed that the elevated miR-24 expression was significantly correlated with poor DFS/PFS/RFS (HR = 1.75, 95% CI: 1.17–2.62) (Figure 3). In addition, we also analyzed DFS and RFS data separately. We found that elevated miR-24 expression predicted poor DFS (HR = 2.31; 95% CI: 1.31–4.07) and RFS (HR = 2.02, 95% CI: 1.02–3.98).

### Elevated miR-24 expression and clinicopathological features

To investigate the relationship between elevated miR-24 expression and clinicopathological features, we extracted clinical data including gender, age, tumor diameter, tumor stage, and lymph node status from the published studies (Table 3). The pooled OR suggested that elevated miR-24 expression was positively correlated with tumor diameter (large or small)(OR: 1.25, 95 CI%: 1.04–2.15) and tumor stage (III–IV vs I–II) (OR: 2.41, 95 CI%: 1.53–2.90).

**Table 3.** Relationship between elevated miR-24 expression and clinicopathological features

Clinicopathologic features	No. of studies	Estimate OR (95%CI)	p-value	Heterogeneity		
				I^2^(%)	*p*-Value	Model
Gender (Male vs Female)	7	1.10 (0.73–1.65)	0.658	0	0.603	Fixed
Age (Old vs Young)	7	0.84 (0.59–1.22)	0.366	0	0.902	Fixed
Tumor diamter (Big vs Small)	5	1.25 (1.04–2.15)	0	0	0.216	Fixed
Tumor stage ((III–IV vs I–II)	4	2.41 (1.53–2.90)	0.002	30.8	0.014	Fixed
Lymph node status (Yes vs No)	2	0.89 (0.42–1.66)	0.595	0	0.765	Fixed

### Sensitivity analysis

**Figure 4.**
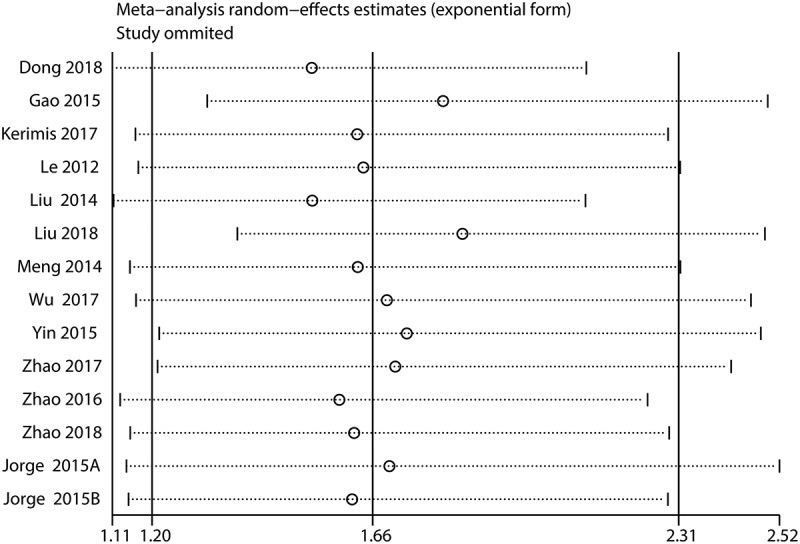
Sensitivity analysis for OS

**Figure 5.**
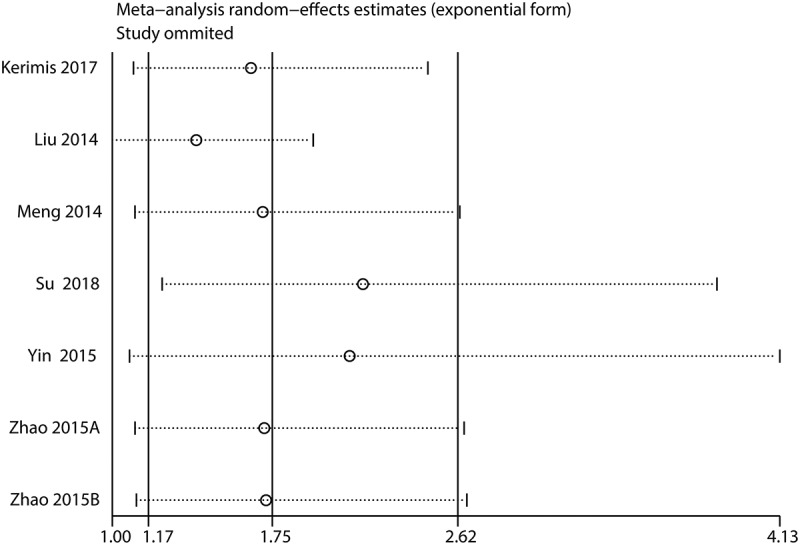
Sensitivity analysis for DFS/PFS/RFS

Sensitivity analysis was conducted by excluding each study in turn to explore the stability of the results. The results did not differ significantly from the overall analysis (Figures 4 and Figures 5), revealing that the outcomes were stable.

### Publication bias

**Figure 6.**
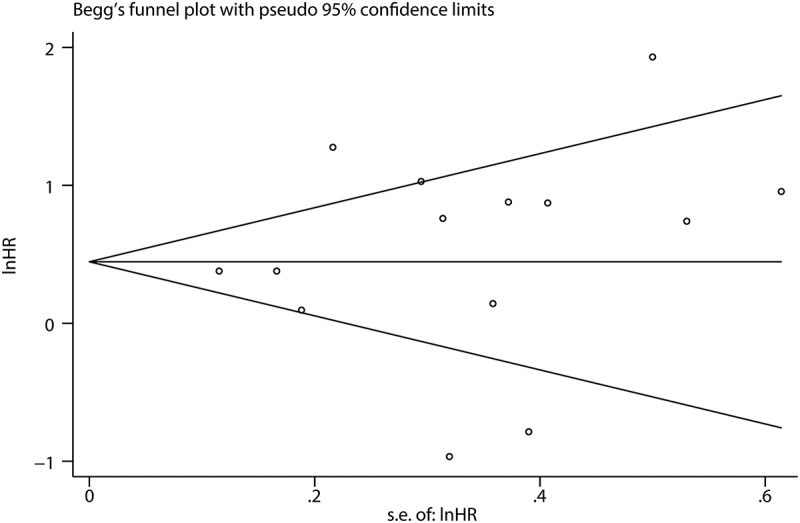
Funnel plots for publication bias for OS

**Figure 7.**
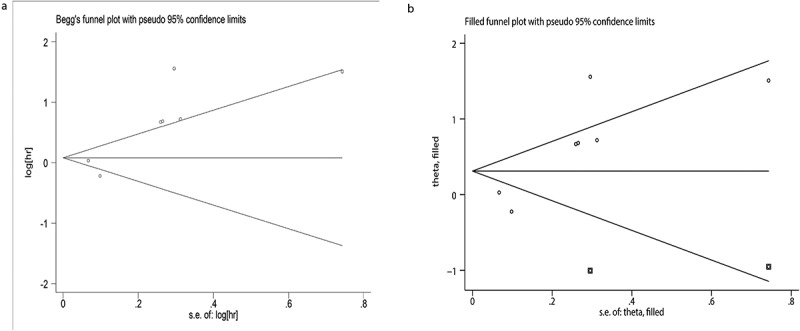
Funnel plots for publication bias for DFS/PFS/RFS. (a) Begg’s test to evaluate DFS/PFS/RFS data. (b) Trim and fill to evaluate DFS/PFS/RFS data

To determine whether there was a publication bias, funnel plots and Egger’s and Begg’s tests were used to test the publication bias. The funnel plots of OS and DFS/PFS/RFS were almost symmetrical. P-values for the Begg’s and Egger’s tests for OS were 0.743 and 0.609, respectively. The P-value was >0.05, which indicated there was no significant bias for OS (Figure 6). The P-values for the Begg’s and Egger’s Tests for DFS/PFS/RFS were 0.548 and 0.028, respectively, indicating that there was a certain bias (Figure 7(a)). Therefore, we adopted the trim-and-fill method for further analysis, and the results showed that the pooled HR for DFS/PFS/RFS was 1.357 (95%CI: 0.914–2.014), which confirmed the results were affected by publication bias (Figure 7(b)).

## Discussion

Cancer is a serious global health problem, which not only imposes a heavy burden on the medical system, but also causes great distress to patients. According to statistics, there were more than 18.1 million new cancer patients and 9.6 million cancer deaths worldwide in 2018 [32]. Advanced tumors are often malignant, prone to relapse after treatment and have poor prognosis. Many diagnostic and prognostic markers have been applied to cancers, but the results may be not satisfactory [33–35]. Thus, it is important to identify more effective prognostic markers.

The relationship between miR-24 and cancer has always been a significant focus of research interest. MiR-24 can regulate tumor progression by degrading mRNA, destroying the stability of targeted mRNAs, or preventing their translation. MiR-24 is considered to act on an indispensable role in tumor development, tumor development, and it may be an effective prognostic marker for tumors. MiR-24 can regulate tumors in many ways. Du et al. found that miR-24 enhanced breast tumor cell invasion and metastasis through THE PTPN9/PTPRF/EGF signaling axis [36]. Roscigno et al. reported that miR-24 inhibits chemotherapy-induced apoptosis of breast cancer stem cells through the FIH1-HIFa signaling pathway, and enhances the resistance of tumor cells to hypoxia [37]. Lin et al. revealed that miR-24 can target P57 to promote the proliferation of oral squamous cell carcinoma cells [38]. Yu et al. disclosed that miR-24 promotes the proliferation, migration, and invasion of bladder cancer cells by inhibiting DEDD [39]. In lung cancer, miR-24 strengthened tumor proliferation and migration by directly targeting SOX7 in tumor cells and in a xenograft mouse model [7]. Dong et al. suggested that miR-24 could restrain liver cancer cell proliferation and accelerate tumor formation by targeting Metallothionein 1 M [40]. In addition, P53 has also been confirmed to be a regulatory gene targeted by miR-24 to promote liver cancer cell metastasis and invasion [41]. Chen et al. showed that miR-24 targeted ST7 through β-catenin/Tcf-4 signaling pathway to enhance the proliferation and invasion of glioma [42]. Liu et al. reported that miR-24 may achieve its biological function by regulating Bim [43]. Moreover, researchers discovered that miR-24 could promote colon cancer cell proliferation, invasion, and migration partially by repressing TRIM11 [5]. It can be seen from the above that miR-24 is responsible for different regulatory mechanisms in different tumors, and may be a potentially valuable target for tumor treatment.

Many studies have shown that miR-24 is abnormally expressed in a variety of tumors and has often been associated with prognosis, but a definitive conclusion has not been reached. Gao et al. explored 95 CRC patients who underwent radical surgery, and found that low miR-24 expression was significantly correlated with local invasion, lymph node metastasis and clinical stage and was a risk factor for poor prognosis [11]. Dong found that elevated miR-24 expression in GC tissue was correlated with the pathological differentiation degree, lymph node metastasis and depth of infiltration [10]. Further multivariate cox regression analysis showed that high miR-24 expression was a favorable prognostic factor in GC patients [10]. Zhao observed that lung cancer patients with high miR-24 expression were remarkably shorter OS [21], and miR-24 expression was correlated with tumor size and tumor node metastasis. In addition, Kerimis et al. revealed that high miR-24 expression predicted poor OS and DFS in patients with colorectal adenocarcinoma, independently of clinicopathological parameters [13]. These different results indicate that miR-24 plays different important roles in different tumors.

We implemented a meta-analysis to comprehensively clarify the prognostic role of miR-24 in different tumors. Our meta-analysis displayed that elevated miR-24 expression was closely correlated with adverse OS and DFS/PFS/RFS. In the subgroup analysis for OS, we found that elevated miR-24 expression was mainly associated with leukemia (1.40 [1.17–1.69]), HCC (3.03 [2.14–4.30]), and LC (2.28 [1.21–4.29]), which indicated that miR-24 may be a better predictor of outcome for these three types of tumors. Interestingly, we also found that regardless of the White or Yellow race, higher miR-24 expression was closely associated with poor prognosis. By comparing elevated miR-24 expression with clinicopathological data, we also found that elevated mir-24 expression was positively correlated with tumor diameter (large vs small) and tumor stage (III–IV vs I–II). Mir-24 may be involved in tumor development by influencing tumor proliferation, invasion, and metastasis. Thus, miR-24 may be a suitable and effective indicator of cancer prognosis.

Some unavoidable flaws exist in this study. Firstly, the analysis was from a handful of publications. Secondly, all enrolled studies were small-sample retrospective studies. Thirdly, most of the included studies were implemented in Asia, which affected the universality of the results. Fourth, some HRs and 95% CI were extracted from the survival curve, which may have led to an interpretation error. Fifth, the results need to be viewed with caution due to the heterogeneity. Finally, we did not compare the analysis with any public domain datasets or consortium with similar claims for miR-24.

Of course, our study has some advantages. Firstly, the sensitivity analysis showed that the meta-analyses were stable. Secondly, no publication bias was observed in the results of pooled OS. In addition, a previous meta-analysis explored the prognostic value of microRNA‑ 23A/24‑2 ‑ 27a, but the prognostic significance of miR-24 was not evaluated in depth because of the limited number of enrolled articles [44]. We further clarify the prognostic significance of miR-24 in tumors by compiling a large amount of literatures.

## Conclusion

Our results indicated that elevated miR-24 expression predicts poor OS. We suggested that miR-24 could be a promising prognostic marker in cancers. Due to inevitable shortcomings, future prospective studies are necessary to verify our findings and further assess the relationship between miR-24 and cancers.

## Data Availability

All data are in the manuscript and can be obtained from the corresponding author.

## Article highlights

1. We revealed that elevated miR-24 expression was significantly associated with poor overall survival in patients with cancers.
2. we found that elevated miR-24 expression was positively correlated with tumor size (large or small) and tumor stage (III–IV vs I–II) in patients with cancers.
3. Elevated miR-24 expression indicates poor prognosis and can serve as an effective prognostic indicator in different cancers.

## Abbreviation

HR, hazard ratio; OR, odds ratio; CI, confidence interval; OS, overall survival; DFS/PFS/RFS, disease-free survival/recurrence-free survival/progression-free survival; GC, gastric cancer; LC, lung cancer; CRC, colorectal cancer; ALL, acute lymphoblastic leukemia; AML, acute myeloid leukemia; HCC, hepatocellular carcinoma; NPC, nasopharyngeal carcinoma; TSCC, tongue squamous cell carcinoma; NOS, Newcastle Ottawa scale;
